# PANSAID—PAracetamol and NSAID in combination: detailed statistical analysis plan for a randomised, blinded, parallel, four-group multicentre clinical trial

**DOI:** 10.1186/s13063-017-2203-1

**Published:** 2017-10-10

**Authors:** Kasper Højgaard Thybo, Janus Christian Jakobsen, Daniel Hägi-Pedersen, Niels Anker Pedersen, Jørgen Berg Dahl, Henrik Morville Schrøder, Hans Henrik Bülow, Jan Gottfrid Bjørck, Søren Overgaard, Ole Mathiesen, Jørn Wetterslev

**Affiliations:** 1 Department of Anaesthesiology, Næstved Hospital, Ringstedgade 61, 4700 Næstved, Denmark; 2 Copenhagen Trial Unit, Rigshospitalet, Dept. 7812, Blegdamsvej 9, 2100 Copenhagen Ø, Denmark; 3 Department of Anaesthesiology, Gildhøj Privathospital, Brøndbyvester Blvd. 16, 2605 Brøndby, Denmark; 4 Department of Anaesthesiology, Bispebjerg Hospital, Bispebjerg Bakke 23, 2400 Copenhagen NV, Denmark; 5 Department of Ortopedic Surgergy, Næstved Hospital, Ringstedgade 61, 4700 Næstved, Denmark; 6 Department of Anaesthesiology, Holbæk Hospital, Smedelundsgade 60, 4300 Holbæk, Denmark; 7 Department of Ortopedic Surgergy, Nykøbing Falster Hospital, Fjordvej 15, 4800 Nykøbing Falster, Denmark; 8 Department of Orthopaedic Surgery and Traumatology, Odense University Hospital, Sdr. Boulevard 29, DK-5000 Odense C, Denmark; 9 Department of Clinical Research, University of Southern Denmark, Sdr. Boulevard 29, DK-5000 Odense C, Denmark; 10 Centre of Anaesthesiological Research, Department of Anaesthesiology, Zealand University Hospital Køge, Lykkebækvej 1, 4600 Køge, Denmark

**Keywords:** Ibuprofen, Paracetamol, Total hip arthroplasty, Benefit, Harm, Multimodal analgesia, Postoperative pain, Detailed statistical analysis plan, Randomised controlled trial

## Abstract

**Background:**

Effective postoperative pain management is essential for the rehabilitation of the surgical patient. The PANSAID trial evaluates the analgesic effects and safety of the combination of paracetamol and ibuprofen. This paper describes in detail the statistical analysis plan for the primary publication to prevent outcome reporting bias and data-driven analysis results.

**Methods/design:**

The PANSAID trial is a multicentre, randomised, controlled, parallel, four-group clinical trial comparing the beneficial and harmful effects of different doses and combinations of paracetamol and ibuprofen in patients having total hip arthroplastic surgery. Patients, caregivers, physicians, investigators, and statisticians are blinded to the intervention. The two co-primary outcomes are 24-h consumption of morphine and proportion of patients with one or more serious adverse events within 90 days after surgery. Secondary outcomes are pain scores during mobilisation and at rest at 6 and 24 h postoperatively, and the proportion of patients with one or more adverse events within 24 h postoperatively.

**Discussion:**

PANSAID will provide a large trial with low risk of bias regarding benefits and harms of the combination of paracetamol and ibuprofen used in a perioperative setting.

**Trial registration:**

ClinicalTrials.org identifier: NCT02571361. Registered on 7 October 2015.

**Electronic supplementary material:**

The online version of this article (doi:10.1186/s13063-017-2203-1) contains supplementary material, which is available to authorized users.

## Background

Effective postoperative pain management is a core component in enhanced recovery after surgery programs and is essential for the rehabilitation of surgical patients [1–3]. Patients are often treated with different combinations of non-opioid drugs and analgesic methods (“multimodal analgesia”) to achieve better analgesic effects and lower the requirement for opioids.

The literature on postoperative multimodal analgesia is characterised by small studies using a variety of different combinations of drugs, inconsistency in outcome reporting, and types of surgery with short follow-up [4, 5]. Consequently, the effects of most combinations of analgesics are not well documented [4] and there is a significant risk that patients’ pain will either be treated insufficiently or that patients receive combinations of analgesics without additive effects but with an increased risk of adverse effects [5].

The International Conference on Harmonisation (ICH) of Good Clinical Practice (GCP) [6] and leading experts recommend that randomised clinical trials should be analysed according to predefined outcomes and a predefined detailed statistical analysis plan [7]. To prevent outcome reporting bias and data-driven analysis results and increase transparency this paper will in detail describe the detailed statistical analysis plan for the PANSAID trial [8] while enrolment of patients and collection of data is still on-going and before the database is accessed for trial results.

## Methods/design

### Trial overview

**Fig. 1:**
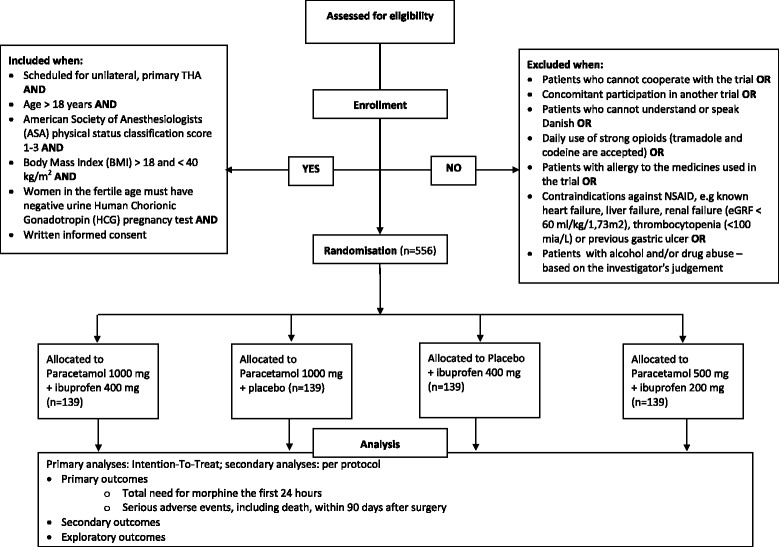
CONSORT Flow-diagram

**Fig. 2:**
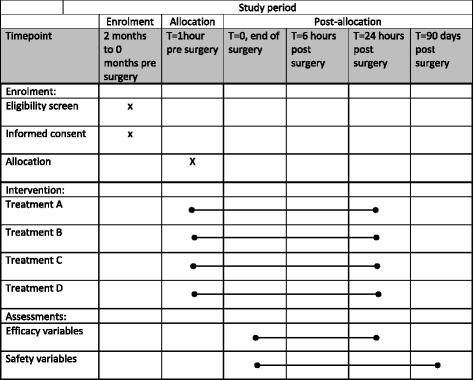
SPIRIT figure: PANSAID

PANSAID is a multicentre, randomised, controlled, parallel, four-group clinical trial comparing the beneficial and harmful effects of different doses and combinations of paracetamol and ibuprofen in patients having total hip arthroplasty (THA) surgery. Patients are randomised to four groups in a 1:1:1:1 ratio (Fig. 1, Additional file 1). The patients will receive one of the following treatments for the first 24 h postoperatively: Treatment A) paracetamol 1 g + ibuprofen 400 mg, four times; Treatment B) paracetamol 1 g + placebo, four times; Treatment C) placebo + ibuprofen 400 mg, four times; Treatment D) paracetamol 0.5 g + ibuprofen 200 mg, four times (Fig. 2). The patients, caregivers, physicians, investigators, and statisticians are blinded to the intervention. Conclusions will be made blinded to the interventions. The patients are enrolled in the trial only after obtaining informed consent. The trial is conducted at six centres (Næstved, Holbæk, and Nykøbing Falster Hospitals, Gildhøj Private Hospital, Odense University Hospital, and Zealand University Hospital Køge). The aim of the PANSAID trial is to investigate analgesic effects and safety of paracetamol and ibuprofen and their combination in different dosages after THA. The trial background, design, and rationale have previously been published [8].

The trial is registered at ClinicalTrials.org (NCT02571361) and EudraCT (2015-002239-16) and conducted in accordance with the Helsinki Declaration and approved by the Biomedical Research Ethics Committee of Region Zealand (SJ-462), Danish Medicine Agency (EudraCT 2015-002239-16), and the Danish Data Protection Agency (REG-33-2015). The progression of the trial can be monitored at the homepage (www.PANSAID.dk).

This detailed statistical analysis plan has been written while data collection from the PANSAID trial is on-going. The data analysis of the main publication will follow this plan. The detailed statistical analysis plan was approved by the PANSAID steering committee on 25 April 2017.

### Sample size

The PANSAID trial has two primary outcomes: 1) 24-h postoperative morphine consumption; and 2) proportion of patients with one or more serious adverse events (SAE) within 90 days after surgery. The sample size estimation is based on 24-h morphine consumption. Due to six possible comparisons and a wish to limit the family-wise error rate to 0.05 (a 0.025 (two-sided) error rate for each of the two primary outcomes), the type one error rate is α = 0.0042 (two-sided). Based on power (1 – β) = 0.90, α = 0.0042, and standard deviation of 20 mg (unpublished data from our research team) we need to randomise 139 individuals in each intervention group (556 in total) to detect a minimal clinically relevant difference of 10 mg in 24-h morphine consumption. We expect the largest difference between Group A (paracetamol 1 g + ibuprofen 400 mg) and Group B (paracetamol 1 g + placebo).

For the co-primary outcome of patients with one or more SAE within 90 days of surgery, we will collate events according to whether the patients used ibuprofen corresponding to one comparison of 417 versus 139 patients. With a type one error rate of 0.025 (two-sided) this renders a power of 80% to detect an increase in patients having one or more SAE within 90 days of surgery from 10% to 21% if ibuprofen is used [9].

This sample size estimation has been made with the power and sample size program PS [10] and is based on the assumption that data on supplementary morphine consumption will be normally distributed. This assumption may not hold and we consider the possibility that a non-parametric test will be necessary if normal distribution by Log transformation is unobtainable. However, we do not expect indefinite tails (very large standard deviations) in the distribution of supplementary morphine consumption and we have many more patients in our intervention groups than required for using explicit sample size calculations for the use of the Van Elteren test [11].

We do not expect that any patients will be lost to follow-up. If the patient-controlled analgesia (PCA)-morphine is discontinued we will convert additional oral/intravenous opioid to morphine equivalent doses (at this point we are missing two morphine values (2/482 = 0.4%)). Regarding the outcome of SAE, the Danish National Patient Registry provides data on all citizens in Denmark and therefore we will only have missing values on the rate of SAE if participants leave Denmark (which is very unlikely so soon after surgery).

### Stratification and design variables

The only stratification variables used in the randomisation is site. All primary analyses will be adjusted for ‘site’. We will secondly adjust analyses for age (continuous), sex (male/ female), previous use of paracetamol (daily use, as needed, or not), and previous use of non-steroidal anti-inflammatory drugs (NSAIDs) (daily use, as needed, or not). Results from the adjusted analyses will be briefly presented in the primary publication (in the text, supplementary material, or as a forest-plot). In-depth analyses of all the sub-groups (and the adjusted analysis) will follow in subsequent publications. The sub-groups have previously been defined [8] and can been seen at the trial website.

### Outcomes

#### Primary outcome

PANSAID has two co-primary outcomes:

- Total 24-h morphine consumption administered as: 1) PCA-morphine pump from end of surgery (2 mg/dose; lockout 10 min); and 2) additional boluses given the first hour after the end of surgery.
- Proportion of patients having one or more SAE from end of surgery to 90 days postoperatively. Serious adverse events are defined as SAE (according to ICH-GCP guidelines) including death, but except for ‘prolongation of hospitalisation’.

#### Secondary outcomes

- Pain scores (visual analogue scale (VAS), 0–100 mm) at rest at 6 and 24 h postoperatively.
- Pain scores (VAS, 0–100 mm) during 30 degree flexion of the hip at 6 and 24 h postoperatively.
- Proportion of patients with one or more adverse events (AE) in the intervention period (the first 24 h postoperatively).

#### Exploratory outcomes

- Level of nausea at 6 and 24 h postoperatively (none, mild, moderate, severe).
- Number of vomiting episodes (0–24 h).
- Consumption of ondansetron (mg) in the period 0–24 h postoperatively.
- Level of sedation at 6 and 24 h postoperatively (none, mild, moderate, severe).
- Level of dizziness at 6 and 24 h postoperatively (none, mild, moderate, severe).
- Blood loss during the surgical procedure (intraoperatively; ml).
- Days alive outside hospital within 90 days after surgery.

### Measurement of outcome variables

Local investigators and clinical staff at each hospital collect outcome data in the intervention period (0 to 24 h postoperative). The data are collected at 6 (± 1) and 24 (± 1) h postoperatively. Pain scores, nausea, vomiting, dizziness, and sedation are reported by the patients. Follow-up data are collected by interviewing the patient (either by telephone or at the hospital if a 3-month follow-up visit is scheduled by the surgeon), and by registries (the Danish National Patient Registry for SAEs and death).

The exact definitions of outcome variables are described in the protocol article [8].

### Baseline characteristics

The baseline characteristics of the participants will be assessed after inclusion in the trial using data from the time of scheduling the surgery to randomisation. The baseline characteristics can be seen in Table 1.

**Table 1:** Baseline characteristics

Demographic characteristics:	Age (years)
	Sex (male/female)
	American Society of Anaesthesiologist (ASA) physical status score
	Height (cm)
	Weight (kg)
	Body mass index (BMI; kg/m^2^)
	Prior use of analgesic medication
	• Use of paracetamol: no use, daily use, or ‘as needed’
	• Use of ibuprofen: no use, daily use, or ‘as needed’
	• Use of tramadol: no use, daily use, or ‘as needed’
	• Use of codeine: no use, daily use, or ‘as needed’
	• Use of other non-steroidal anti-inflammatory drugs: no use, daily use, or ‘as needed’
Surgical characteristics:	Duration of surgery (min)
	Type of surgery
	• Uncemented, cemented, or hybrid total hip arthroplasty
	Type of anaesthesia
	• General anaesthesia, spinal anaesthesia, conversion of spinal anaesthesia to general anaesthesia, or spinal anaesthesia with sedation ○ If general anaesthesia: amount of sufentanil (μg) given 15 min before ‘end of surgery’ ○ If spinal anaesthesia: amount of bupivacaine (mg) used

### General analyses principles and populations

The primary conclusion of the trial will be based on the results from the primary analyses of the co-primary outcomes. All analyses will be made on both a modified intention-to-treat (mITT) population and a ‘strictly per protocol’ (sPP) population. The mITT population will include patients randomised and having the THA surgery (excluding patient randomised, but afterwards cancelled in the operating room). The sPP population will exclude patients having one or more major protocol violations. Major protocol violations are defined as:

1. Patients that did not get any of the dosages of the randomized allocated trial treatment, *or*
2. Patients withdrawing from the trial intervention allowing the use of registered data, *or*
3. Patients undergoing additional surgery (besides the elective THA) *or* a procedure in the intervention period that requires anaesthesia or sedation and/or analgesia

The sPP analyses regarding SAEs and other safety variables will, however, include patients with major protocol violation definition number 3. Furthermore, the sPP population will exclude patients who received any analgesic medications in the intervention period other than the study medication and PCA-morphine (e.g. paracetamol, NSAIDs, intrathecal opioids, steroids, local infiltration analgesia, etc.). The evaluability of each patient for the statistical analyses will be performed before the code is broken.

If patients have received opioids other than PCA-morphine in the intervention period these will be converted to morphine equivalents (according to Appendix 2 in Hojer Karlsen et al. [12]) and added to the cumulated dose of consumed PCA-morphine.

The trial is conducted at six sites. There are no fixed sample size requirements for each site. We will merge sites with fewer than 30 patients when entering the site as a co-variable in the statistical models. We expect to merge the sites Holbæk and Nykøbing Falster Hospitals. These two hospitals are similar with respect to size, proportion of elective surgery compared with emergency surgery, etc.

### Level of significance

To maintain an overall family wise error rate of 0.05 the level of significance for the co-primary outcome regarding 24-h morphine consumption has been set to 0.0042 (two-sided) after a Bonferroni adjustment due to two co-primary outcomes and six possible comparisons and the anticipation that the intervention effect estimated from these comparisons do not correlate. The level of significance for the co-primary outcome regarding SAE has been set to 0.025 (two-sided) after a Bonferroni adjustment due to two co-primary outcomes anticipated to be independent. We have corrected the secondary outcomes because of the six comparisons and the levels of significance for the secondary outcomes are 0.0084 (two-sided). Due to the hypothesis generating nature of the exploratory outcomes these will not be corrected for multiple comparisons and the level of significance is 0.05 (two-sided).

### Handling of missing data

If data are only missing on the dependent variable then we will use the complete cases only. Otherwise, if there are more than 5% missing data and Littles’s test is statistically significant we will use multiple imputations (MI) to impute missing data [13, 14]. Complete case analysis will be performed as well but the results of the analyses using MI imputed datasets will be considered the primary result of the trial.

### Statistical analysis

#### Statistical analysis of the primary outcome

The primary analysis of the continuous outcome of morphine consumption within 24 h will be pair-wise comparisons between the median consumption of morphine between the four groups (six analyses) adjusted for sites based on the mITT population. We will use Van Elterens test [15] to stratify for site. We will use bootstrapping to present 99.6% and 95% confidence intervals for the mean difference.

The secondary analyses of the primary outcome of morphine consumption are:

- An analysis adjusted for sites based on the sPP population.
- An analysis using the generalized estimation equations (GEE) [16] based on the mITT. This analysis will be adjusted for ‘site’ and design variables (age (continuous), sex (male/ female), previous use of paracetamol (daily use, as needed, or not), and previous use of NSAID (daily use, as needed, or not).

If the outcome of morphine consumption is normally distributed, or normally distributed when log-transferred, we will use conventional parametric statistics. However, we expect the distribution of this outcome to be non-normal.

The primary analysis of the proportion of patients with one or more SAE in the trial period (within 90 days after surgery) will be analysed using GEE with a log link function to obtain relative risks (RRs), combining the groups receiving ibuprofen (417 patients) and comparing them to patients not receiving ibuprofen (139 patients) using the mITT population adjusted for site.

Secondary analyses of number of patients with one or more SAE are:

- An analysis adjusted for ‘site’ based on the sPP population
- GEE adjusted for ‘site’ and design variables based on the mITT population.

As a sensitivity analysis of the outcome of SAE we will perform an analysis excluding patients from Group B (paracetamol + placebo) who took NSAIDs in the follow-up period. This is not described in detail in the protocol. However, our main objective is to investigate whether the use of NSAIDs in the in-hospital period (the intervention period) increases the numbers of SAE despite patients using these drugs when discharged from hospital.

We have chosen the GEE model because of its ability to handle correlated data and few events per site. GEE is a valid method that can be used both for analysis of longitudinal data as well as intra-centre correlations [17]. For all GEE models we will use model-based (non-robust) standard errors [16] and site will be entered as a clustering variable.

#### Statistical analysis of the secondary outcomes

The primary analysis of the continuous data of pain scores are analysed using GEE adjusted for ‘site’.

The primary analysis of the variable of the proportion of patients having one or more AE in the intervention period will be analysed using GEE with a log link function to obtain RRs adjusted for ‘site’.

Secondary analyses of the secondary outcomes are:

- Analyses adjusted for ‘site’ based on the sPP population
- Analyses adjusted for ‘site’ and design variables based on the mITT population using GEE for the continuous data of pain scores and a GEE with a log link function for the variable of patients having one or more AE in the intervention period.

#### Statistical analysis of the exploratory outcomes

For the exploratory outcomes of nausea, dizziness, and sedation, we will dichotomize data to none/mild versus moderate/severe and analysed using GEE with a log link function adjusted for ‘site’ to obtain RRs.

The continuous outcomes of consumption of ondansetron, number of vomiting episodes, days alive and outside hospital, and blood loss during the surgical procedure will be analysed using the van Elteren test adjusted for ‘site’.

Secondary analyses of the exploratory outcomes are:

- Analyses adjusted for ‘site’ based on the sPP population

### Outline of figures and tables

The first figure will be a Consolidated Standards of Reporting of Randomised Trials (CONSORT) flow chart. The first table will be the baseline characteristics of the mITT population. The second table will be of the two co-primary outcomes (24-h morphine consumption and number of patients with one or more SAE within 90 days) according to the four groups and pair-wise comparisons. The third table will be the secondary and exploratory outcomes.

### Blinding of the statistician

Prior to breaking of the randomisation code an independent statistician will perform the data analyses according to this detailed statistical analysis plan. The analyses of the SAE outcome will be between Group B and all other groups combined. In order to maintain blinding the statistician will perform the analyses of the SAE outcome in four rounds assuming that one of the groups is Group B.

Based on the masked result, the steering committee will agree upon abstracts covering all possible combinations and then the blinding will be broken [18]. The final manuscript will contain the correct pre-written abstract.

## Discussion

PANSAID will provide data from a large trial with overall low risk of bias regarding benefits and harms of the combination of paracetamol and ibuprofen used in a perioperative setting, which is urgently needed [19, 20]. The full, pseudo-anonymised data set will be made publicly available 18 months after last follow-up of the last randomised patient.

## Trial status

At present, more than 500 patients have been enrolled in the trial and we expect to finish recruiting in October 2017.

## Additional file

Additional file 1: CONSORT checklist. (DOC 122 kb)

## Abbreviations

AE: Adverse events
CONSORT: Consolidated Standards of Reporting of Randomised Trials
GCP: Good Clinical Practice
GEE: Generalized estimation equations
ICH: International Conference on Harmonisation
MI: Multiple imputations
mITT: Modified intention-to-treat
NSAID: Non-steroidal anti-inflammatory drug
PCA: Patient-controlled analgesia
RR: Relative risk
SAE: Serious adverse events
sPP: Strictly per protocol
THA: Total hip arthroplasty
VAS: Visual analogue scale
